# The occurrence of osteoarthritis at a minimum of ten years after reconstruction of the anterior cruciate ligament

**DOI:** 10.1186/1749-799X-3-24

**Published:** 2008-06-10

**Authors:** Cor P van der Hart, Michel PJ van den Bekerom, Thomas W Patt

**Affiliations:** 1 Department of Orthopaedic Surgery, Onze Lieve vrouwe Gasthuis, Oosterpark 9, Postbus 95500, 1090 HM, Amsterdam, the Netherlands; 2 Department of Orthopaedic Surgery, Academic Medical Center, Meibergdreef 9, Postbus 22660, 1105 AZ, Amsterdam, the Netherlands; 3 Department of Orthopaedic Surgery, Sint Maartenskliniek, Polanerbaan 2, Postbus 8000, 3440 JD, Woerden, the Netherlands

## Abstract

**Objective:**

The objective of this study was to evaluate the incidence of radiographic osteoarthritis in the operated knee in comparison with the contralateral knee ten years after a bone-tendon bone patellar autograft ACL-reconstruction and to evaluate to which level patients regain activity ten years after reconstruction.

**Methods:**

Fifty-three patients with ACL instability were operated arthroscopically using the central third of the patellar tendon as a bone-tendon-bone autograft. At a minimum of 10 year follow up 28/44 patients matched the inclusion criteria and could be reached for follow-up. Evaluation included a patient satisfaction evaluation using a Visual Analog Scale, physical examination (International Knee Documentation Committee score, Tegner score, Lysholm score, KT-1000 stabilometry) and a radiological evaluation (Kellgren and Fairbanks classification).

**Results:**

The patients' satisfaction, at a mean of 10,3 year follow-up, measured with a VAS score (0–10) was high with a mean of 8.5 (range 4 to 10). The KT 1000 arthrometer laxity measurements revealed in 55% of the patients an A rating (1–2 mm), in 29% a B rating (3–5 mm) and in 16% a C rating (6–10 mm). According to the Tegner score 54% of the patients were able to perform at the same activity level as pre-operatively. The mean pre-operative Tegner score was 6.8 and the mean post-operative Tegner score was 6.0 at final follow up. The Lysholm score showed satisfactory results with a mean of 91 points (range 56 to 100). According to the Kellgren and Fairbank classifications, there is a significant difference (p < 0.05) in development of OA between the ACL injured and subsequently operated knee in comparison to the contralateral knee.

**Conclusion:**

The patellar BTB ACL reconstruction does not prevent the occurrence of radiological OA after 10 years but does help the patient to regain the pre-operative level of activity.

## Background

The anterior cruciate ligament (ACL) is one of the most frequently injured ligaments in the human body. Estimated incidences of 0.24 to 0.34 ACL injuries per 1000 population per year have been reported [1-3]. Some authors [4,5] made an estimation of 250,000 ACL injuries per year worldwide. Anterior cruciate ligament injury frequently affects young active people with long working and sporting futures. The importance of the ACL to the normal knee function has been emphasized by many investigators. The ACL is the primary stabiliser against anterior translation of the tibia on the femur and is important in counteracting rotation and valgus stress. In activities which demand pivoting, cutting and side stepping, such as soccer, rugby and field hockey deficient function of the ACL leads to rotational laxity. This results in recurrent injuries and increased risk of intra-articular damage, inclusion meniscal tears and degenerative changes. Disruption of the ACL often leads to significant disability which can lead to changes in lifestyle. Although both operative and non-operative treatments have been proposed, randomised controlled trials (RCT) have shown the superiority of reconstruction compared with primary repair [6,7]. Additional RCT's have shown no clinical differences between the use of patellar tendon autograft and the use of hamstrings tendon autograft [8-15] or between the one or two incision arthroscopic operative techniques [14,16-18]. In the data of 292 patients presented by Daniel et al. [1] the management decisions were made by the patients and their treating orthopaedic surgeons. Patients who did not elect for early ACL reconstruction were directed in a home exercise program [19]. The patients were advised not to participate in running sports for three months after injury until the range of motion (ROM) was full and there was no effusion. They was advised not to participate in jumping, pivoting, hard cutting and lateral motion sports for a minimum of three months. After this a brace was advised during sport activities for those with unstable knees. After a period of 6 months of rehabilitation, the patients who could not participate in their favourite sport activities, due to anterior knee instability or repeating giving way episodes, ACL reconstructive surgery was advised. Daniel concluded that there is a low probability that patients with an acute traumatic haemarthrosis that is found stable on instrumented examination will develop laxity over a five year period and that many of the ACL injured patients who did not undergo ACL reconstruction surgery continued to participate in sports activities.

The use of a patellar bone-tendon-bone (BTB) graft seems to be favoured by surgeons, especially when dealing with athletes involved in contact sports [20]. Practice patterns throughout the world vary in the timing of reconstruction in anterior cruciate ligament deficient knees. A debate continues regarding whether reconstruction should be performed early before onset of instability episodes or be delayed until the patient has shown that rehabilitation alone is insufficient to maintain normal knee function. [1] Other researchers have highlighted the importance of preserving menisci to prevent early osteoarthritis (OA) in isolated meniscal injuries [21-24]. However, few studies have addressed the results of meniscal preservation in anterior cruciate ligament deficient or reconstructed knees [25,26]. Many studies in this area are flawed by their retrospective nature and hindered by evaluation of outdated open reconstruction techniques.

The primary objective of this study was to evaluate the incidence of radiographic osteoarthritis in the operated knee in comparison with the contralateral knee ten years after a bone-tendon bone (BTB) patellar autograft ACL-reconstruction. The second objective was to evaluate to which level patients regain activity ten years after reconstruction.

## Patients and Methods

### Patient selection

Between March 1993 and January 1994 53 patients with ACL instability were operated arthroscopically using the central third of the patellar tendon as a BTB autograft. The indication for operation was instability secondary to rupture of the ACL confirmed by clinical examination (Lachman grade 2 to 3 and positive Pivot-shift test). These patients were considered at high risk of further knee injury due to the degree of laxity and the desired level of activity [27]. In order to minimise the development of arthrofibrosis, reconstruction was carried out only after the patient had regained a minimum 100° of flexion with minimal effusion or discomfort.

Patients with knee ligament surgery at the contralateral side, ipsilateral revision operation, ipsilateral posterior cruciate ligament (PCL) or posterolateral corner injury, at the time of the first operation, an abnormal radiograph of the knee before the operation and patients who had a total knee arthroplasty (TKA) after 10 years follow-up were not included. Patients who had anterior cruciate ligament deficient knees at the contralateral side were excluded from this study. Forty-four patients matched the including criteria. At a minimum of 10 year follow up 64% of the patients who matched the including criteria (N = 28) could be reached for follow-up. There were 11 women and 17 men. The mean age at the time of surgery was 30.5 years (range 16 to 42).

The left side was involved in 58% and the right in 42% of the patients. The ACL was reconstructed within a mean of 34 months (range 14 to 186) of injury.

### Operative Technique

All procedures were carried out by the senior author. Under general or spinal anesthaesia a single dose intravenous cefamandol (1 × 1500 mg) was administered pre-operatively. A high thigh tourniquet preventing blood loss and optimising view was applied. A diagnostic arthroscopy was undertaken if needed several weeks before the ACL reconstruction and a meniscus suturing or a meniscectomy was done if required.

We perform a reconstruction using a two small incision technique as described by McGuire to prevent scarring of Hoffa's fatpad and to reduce the incidence of donor site morbidity. An arthroscopically assisted technique using the middle third of the patellar tendon with trapezoidal bone block (20 to 25 mm long) (autograft) harvested with two vertical incisions were used. The distal entry of the tibial tunnel is positioned through the distal aspect of the incision near the tibial tubercle [28]. The femoral tunnel was drilled through the tibial tunnel.

The autograft was fixed with a poly L-lactic acid (PLLA) canulated interference screw at the proximal and distal point of graft. (Linvatec, Largo, FL femur 20 of 25/7 mm, tibia 7 of 8/20 of 25 mm). No supplementary fixation was used [28].

Full extension of the knee was ensured before insertion of the tibial screw. Laxity was checked using the anterior drawer and Lachman tests. The patients were in hospital for a mean of 3.5 days with a maximal of 5 days after surgery.

### Post-operative management

The postoperative protocol was uniform for all patients. Immediately post-operatively the knees were subjected to continuous passive motion (CPM) gradually increasing to achieve a ROM of 0° to 90° before discharge. Weight bearing as tolerated was allowed using an extension lock brace. A rehabilitation programme was started on the first post-operative day with closed chain exercises, leading to proprioceptive and sports training after three to six months. Patients were discouraged from returning to competitive sport involving jumping, pivoting or sidestepping until six to nine months after reconstruction and then only after formal clinical evaluation.

### Evaluation

All patients were examined by one independent examiner after ten years of follow-up. Evaluation included a patient satisfaction evaluation using a Visual Analog Scale (VAS), physical examination (International Knee Documentation Committee (IKDC) score, Tegner score, Lysholm score, KT-1000 arthrometer) and a radiological evaluation (Kellgren and Fairbanks classification).

The patients scored their satisfaction with the post-operative result on a scale from 0 (very dissatisfied) to 10 (very satisfied).

The symptoms and signs of knee function were assessed to complete the IKDC knee grade [29]. IKDC grades incorporate multiple subjective and objective criteria. These patients were graded as normal (A), nearly normal (B), abnormal (C) or severely abnormal (D). The final grade is determined by the worst score in any of the four principal categories: subjective assessment, symptoms, ROM and ligament examination. The IKDC grades activity into three categories [29]; these are the level of activity (1. strenous; 2. moderate; 3. light; 4. sedentary), the level of competition, (competitive, vigorous recreational, light recreational, activities of daily living (ADL)) and the total number of hours spent each year at the highest level of activity. There is evidence that the final IKDC grade is reliable compared with other rating scales [30].

The modified Tegner activity score, which levels from 1 to 10, describes increasing demands for the knee according to different types of sport [31].

The Lysholm knee scale [32] is designed to evaluate specific symptoms relating to knee function including limp (5 points), support (5 points), locking (15 points), instability (25 points), pain (25 points), swelling (10 points), stair climbing (10 points) and squatting (5 points).

Instrumented laxity testing was undertaken using the KT1000 stabilometry (MEDMetric Corporation, San Diego, California, US). The relaxed limbs are supported in 30° flexion. The patellar sensor pad is stabilised and the testing reference position is established by pushing with an 89 N load posteriorly and then releasing the load. While the patellar sensor is stabilised with one hand, the other hand applies a strong anterior displacement of force directly to the calf to produce a maximum anterior displacement that is measured by the patellar sensor. The displacement is compared with the contralateral side [33].

Weightbearing antero-posterior (AP), lateral, and femoral-patellar in 30° flexion radiographs were taken of both knees (ipsi- and contralateral) at 10 years. The radiographs were taken under standardised conditions to obtain reproducible images. The grade of OA was evaluated by two independent unbiased blinded radiologists according to the classifications of Kellgren [34] (Table 1) and Fairbank [35] (Table 2).

**Table 1:** Kellgren classification ^46^

**I**	**doubtful**	minute osteophytes, doubtful significance
**II**	**minimal**	definite osteophytes, unimpaired joint space
**III**	**moderate**	moderate diminution of joint space
**IV**	**Severe**	joint space greatly impaired with sclerosis of subchondral bone

**Table 2:** Fairbanks classification ^26^

**Radiological signs**	**Grades**
**1. **Spurring of the tibial spines	**I **no changes
**2. **Marginal osteophytes	**II **one symptom
**3. **Flattening of femur/tibia	**III **two or three changes
**4. **Narrowing of the joint space	**IV **all four changes

### Statistical Analysis

This was undertaken using Microsoft Excel to collect the data. Comparisons between the results at a minimum of ten years follow-up were made using the non-parametric Mann Whitney two-tailed U test. Nonparametric correlations of the remaining laxity with the grade of OA were calculated with the Spearman's rank correlation coefficient (rho). A level of significance of p < 0.05 was used to judge significance.

## Results

The patients' satisfaction, at 10.3 (10–11) year of follow-up, measured with a VAS score (0–10) was high with a mean of 8.5 (range 4 to 10).

The overall IKDC score at the ten year follow-up demonstrated that 36% of the patients rated A, 50% B and 14% C.

According to the Tegner score 54% of the patients were able to perform at the same activity level as pre-operatively, 7% improved one level. 14 % decreased one level, another 14% two levels and 11% three levels. The mean pre-operative Tegner score was 6.8 and the mean post-operative Tegner score was 6.0 at final follow up.

The Lysholm score showed a mean of 91 points (range 56 to 100 points). The patient with Lysholm 56 was the same who had VAS 4.

The KT 1000 arthrometer revealed in 55% of the patients A (1–2 mm), in 29 % of the patients B (3–5 mm) and in 16% of the patients C (6–10 mm) stability.

Looking at the osteoarthritis rate of the operated knee, 55 % of the patients decreased one grade according to the Kellgren classification, 32% of the patients decreased two or more grades, however in 13 % of the patients there was no change compared to the contralateral side. (Table 3 and 4)

**Table 3:** Radiological results according to the Kellgren Classification

	**Ipsilateral**	**Contralateral**	**Difference**
**0**	10%	71%	-61%
**I**	45%	26%	+19%
**II**	29%	3%	+26%
**III**	10%	0%	+10%
**IV**	6%	0%	+6%

**Table 4:** Differences in Kellgren and Fairbank classification

**Grades**	**0**	**+1**	**+2**	**+3**
**Kellgren**	13%	55%	26%	6%
**Fairbanks**	10%	52%	35%	3%

The Fairbank classification showed an increase in osteoarthritis of 1 grade in 52% of the patients, 35% of the patients had an increase of 2 grades and 3% of the patients had an increase of 3 grades. (Table 4, 5 and 6) 10 % of the patients had no changes after 10 years of follow-up. According to both radiological classifications, there is a significant difference (p < 0.05) in development of OA between the ACL injured and subsequently operated knee in comparison to contralateral knee.

**Table 5:** Radiological results according to the Fairbank Classification

	**Ipsilateral**	**Contralateral**	**Difference**
**I**	10%	74%	-64%
**II**	32%	23%	+9%
**III**	48%	3%	+45%
**IV**	10%	0%	+10%

**Table 6:** Radiological Signs according to the Fairbank Criteria

**Radiological signs**	**Ipsilateral**	**Contralateral**
**Spurring of tibial spines**	16	4
**Marginal osteophytes**	23	5
**Flattening of fem & tib**	13	2
**Narrowing of joint space**	5	1

Thirteen patients had a medial meniscal injury, 7 patients had a lateral meniscal injury and all these 7 patients had a combination of medial and lateral meniscal injury, 1 patient had chondral injury grade II, one patient had chondral injury grade III and 2 patients had chondral injury grade IV. In 3 lateral and 1 medial meniscal injuries a suturing was performed. None of the patients developed arthrofibrosis. Six patients required an additional arthroscopy of the operated in the follow-up period. There were no revision operations for failed grafts at 10 year follow-up.

No significant correlation was observed between the remaining laxity (KT-1000) and the grade of OA. (Kellgren and Fairbanks score of the operated knee, difference in Kellgren and Fairbanks score between operated and contralateral non operated knee)

## Discussion

This study evaluates the incidence of radiographic knee osteoarthritis in comparison to the contralateral knee, 10 years after a bone-tendon bone (BTB) patellar autograft ACL reconstruction. The long term effect of ACL reconstruction requires documentation to provide surgeons with a rationale for treatment protocols. This may help surgeons to prognosticate long term effects and educate patients regarding future use of their knees. ACL reconstruction techniques and the rehabilitation programs have evolved rapidly in the past decade. These changes were made with the objective to improve function and ROM post-operatively. These recent changes require additional research to clarify the long term prognosis of the current surgical and rehabilitation techniques. For this reason Lohmander proposed a national register of reconstructive procedures for ACL reconstruction [36].

With a satisfaction VAS of 8.5, the patients are content with the post-operative result of the ACL reconstruction compared to the contralateral side.

Concerning the IKDC grade, Irrgang [30] stated that it may be better to consider knees of grade A and B as one group and those of grade C and D as another. This helps to delineate the abnormal results found in grades C or D. In our study 86% of the patients are in the first group (IKDC A and B) at 10 year follow-up. According to Jomha et al. [37] there is no relationship between the IKDC grade and the post-operative levels of activity. This suggests that even people with stable and symptom free knees do not necessarily return to pre-trauma activities and those changes in individual preferences may account for some modifications in level of activity.

Documenting pre- and post-injury sports activity is an important part of the patient evaluation because disability for sports after ACL injury is the principle reason that patients request ACL reconstruction [1]. One problem with evaluation of knee function, symptoms and activity is that different scores influence each other. The Lysholm score in the present study revealed a mean value of 91 points, but if the ACL reconstructed knee is not challenged by demanding activity, cutting and pivoting sports performance, the score may appear too high, and will reflect the actual function of the knee as well as the satisfaction of the patient. The Lysholm score has never been validated for the purpose of following ACL laxity in spite of its widespread use and that it has problems with a ceiling effect.

We found only a slight decline in activity and sports performance, as specified in a drop in Tegner score from 6.8 to 6.0. In patients who decreased three levels this was all due to non-knee-related reasons. After the follow-up the patients are 10 years older and logically most patients are less active and perform less sport.

Clinical evaluation of anterior displacement and anterior endpoint with the Lachman test has been used to diagnose the ACL disruption with test sensitivity ranging from 73% to 99% [38-40]. There remains a controversy about the usefulness of the KT-1000 as a device to measure the anterior-posterior displacement and to diagnose an ACL disruption. Daniel [41] postulates that there is a 98% probability that a KT unstable knee had an ACL disruption. To avoid false measurements, careful instrument positioning/placement, patella stabilisation, and patient relaxation is required [41,42]. In our study we found that 45% of the patients had a greater anterior-posterior displacement on the operated knee than on the contralateral knee at 10 years follow-up.

The surgical procedure for reconstruction of the ACL may be of importance regarding the risk of eventually developing knee OA. The major factor with the potential to diminish this risk is improvement and maintenance of joint stability, resulting in a lower frequency of repeat injuries, especially of the meniscus. In this study no correlation between the remaining instability measured by the KT-1000 arthrometer and the grade of OA 10 years after the ACL construction was observed.

On the other hand, operative trauma with haemarthrosis, and the occasional necessity for repeated operations, may increase the risk of developing OA. Another factor of possible importance might be the required tension of the graft and the post-operative rehabilitation programme. It has been shown that over-tensioning of the graft can cause changes in knee joint kinematics that may lead to knee OA in the long term [43,44]. Post-operative arthrofibrosis with decreased ROM may also increase the risk for knee deterioration especially in the patellofemoral joint.

The association between meniscectomy and OA has been well documented [45-49]. Medial meniscectomy is more often associated with severe radiologically demonstrable degenerative changes than lateral meniscectomy [37]. Meniscectomy diminishes the joint contact surface area and increases stresses on the tibia [50]. A number of studies have shown that protection of the injured meniscus at the time of ACL reconstruction may be the best chance of slowing down or preventing osteoarthritis in the knee [51,52]. Leaving meniscal tears untreated has not been found to cause any clinical symptoms after ACL reconstruction with a medial follow-up of 2.6 years [53].

Several studies have demonstrated that a higher age at injury or onset of symptoms after knee injury is associated with an increased progression rate of OA [21,54-57]. Yet, several of these reports fail to present adequate data on the age of the patients at time of the injury. In this study a subgroup analysis to evaluate differences in outcomes measurements for different ages at time of ACL rupture was not realistic

Endogenous factors may be contributing to the development of OA and will cause further variation in the frequency of post-traumatic OA after ACL reconstruction. It was shown that patients with meniscectomy who had an endogeneous risk factor for primary OA, reflected by distal interphalangeal OA had a higher frequency of knee OA than patients without this sign [58]. Other endogenous risk factors may be present in the form of genetic variability in the structure of the gene of cartilage type II collagen [59,60].

Osteochondral lesions and osteoarthritis in young patients are often caused by chronic knee instability and varus or valgus malalignment. These knees can be sufficiently treated by osteotomy and cruciate ligament reconstruction at the same time, suggesting that unicompartimental decompression and treatment of instability is a causal and cost-effective therapy delaying the progression of osteoarthritis and minimizing clinical symptoms [49,61]. People with abnormal joint anatomy or alignment, previous joint injury or surgery, joint instability or above average body weight also appear to be at a greater risk of developing osteoarthritis [62].

An increase in frequency of joint changes with increasing time after the injury has been noted in several reports [23,26,46,56,63,64] while others have failed to confirm this observation [21,54,65]. This variability may be explained by the fact that not all cases of knee OA progress [66,67]. It may also due to the heterogeneous study groups and the use of outcome measurements of low precision and reproducibility.

Many reports have noted different frequencies of OA, depending of which criteria were used to define the presence of OA on radiographics. To undermine this problem we used two scales to classify the post-traumatic OA of the knee. Clearly the method used to evaluate the radiographic OA has a significant influence on the apparent outcome of the study. Using two radiographic scales yielded no different conclusions. Daniel described radiographic OA changes (own classification) in 50% of the ACL-injured knees after 5 years. These changes were even more frequent in surgically than conservatively treated patients [1]. Since many studies use different radiographic scales, different clinical outcome measurements and different follow-up periods the results of the studies are difficult to compare [68].

In this study ACL reconstruction was not able to prevent radiological knee OA despite the fact that the patients with the most severe osteoarthritis, the patients who received a knee arthroplasty, were excluded in this study. This seems substantiated in the meta-analysis of 33 studies by the apparent inability of repair or reconstruction of the ACL to delay the progression of OA after knee injury [36]. The question remains whether continued activity on the same or slightly lower sports level is recommended after ACL injury. Roos [69] and Sommerlath [70] found a higher OA rate in very active patients. However the cause and effect relationship is unclear and therefore no conclusion can be drawn about the outcome for an individual who changes activity.

As already proposed by Daniel [1] five possible explanations for the development of OA in the reconstructed knee are:

1. Greater injury in the reconstructed knee before surgery than in the patients who did not choose reconstruction

2. Joint injury occurring at the time of surgery

3. The joint's response to stress deprivation after surgery [71]

4. Prolonged joint inflammation after surgery [72,73]

5. Abnormal joint mechanics after surgery [73]

Our study had several limitations such as the retrospective character of our study; almost all studies that evaluate the development of knee OA after knee ligament surgery are retrospective because of the complexity of the injured knees with different types of tears in stable and unstable knees that make prospective trials difficult to perform. Twenty of the 28 patients had associated meniscal injuries. For the exact incidence of knee OA after ACL reconstruction surgery the results have to be compared with knee OA after meniscectomy in patients with intact ligaments and after isolated injuries to ligaments other than the ACL. The osteoarthritis in the ACL reconstructed can be due to the already mentioned associated intra-articular injuries but the osteoarthritis can also be developed in the period between trauma and reconstruction due to an unstable environment.

The surgery was performed by only one orthopaedic surgeon at our hospital which limits the extrapolation of the findings to other orthopaedics departments. The group of patients is relatively small and we did not mention the injury mechanism.

The strength or our study includes the long term follow-up, the use of validated outcome measures, the use of patient based and objective measurements, the comparison with the non- injured contralateral knee, the fact that the patients were operated in a relatively short interval of time, the patients were evaluated by an independent unbiased investigator, the evaluation of the standardised radiographics is done without the knowledge of patient identity. We agree with Lohmander that the time is right for a national register of reconstructive procedures for ACL reconstruction. This could assist in the identification of suitable procedures and ensure good quality [36].

## Conclusion

The patellar BTB ACL reconstruction performed 34 months after trauma does not prevent radiological OA but does help the patient to regain the pre-operative level of activity despite the anterior-posterior instability at 10 year follow-up. Patients are satisfied with the result of the ACL reconstruction at follow-up.

## Abbreviations

ACL: Anterior Cruciate Ligament; RCT: Randomised Controlled Trial; BTB: Bone-Tendon-Bone; PCL: Posterior Cruciate Ligament; OA: Osteo-arthritis; TKA: Total Knee Arthroplasty; CPM: Continuous Passive Motion; ROM: Range of Motion; VAS: Visual Analog Scale; IKDC: International Knee Documentation Committee; ADL: Activities of Daily Living; AP: Antero-Posterior; PLLA: Poly L-Lactic Acid

## Authors' contributions

CPvdH performed the anterior cruciate ligament reconstructions and participated in the design of the study, MPJvdB wrote the initial manuscript and performed the statistical analyses, TWP designed the study protocol and coordinated the data collection. All authors read and approved the final manuscript.

## References

[B1] Daniel DM, Stone ML, Dobson BE (1994). Fate of the ACL-injured patient. A prospective outcome study. Am J Sports Med.

[B2] Miyasaka KC, Daniel DM, Stone ML, Hirshman P (1991). The incidence of knee ligament injuries in the general population. Am J Knee Surg.

[B3] Nielsen AB, Yde J (1991). Epidemiology of acute knee injuries: A prospective hospital investigation. J Trauma.

[B4] Bobic V (1992). Current concepts in anterior cruciate ligament reconstruction. Surgery.

[B5] Frank CB, Jackson DW (1997). The science of reconstruction of the anterior cruciate ligament. J Bone Joint Surg.

[B6] Andersson C, Odensten M, Good L, Gillquist J (1989). Surgical or non-surgical treatment of acute rupture of the anterior cruciate ligament. A randomized study with long-term follow-up. J Bone Joint Surg Am.

[B7] Engebretsen L, Benum P, Fasting O, Mølster A, Strand T (1990). A prospective, randomized study of three surgical techniques for treatment of acute ruptures of the anterior cruciate ligament. Am J Sports Med.

[B8] Anderson AF, Snyder RB, Lipscomb AB (1994). Anterior cruciate ligament reconstruction using the semitendinosus and gracilis tendons augmented by the losee iliotibial band tenodesis. A long-term study. Am J Sports Med.

[B9] Aune AK, Holm I, Risberg MA, Jensen HK, Steen H (2001). Four-strand hamstring tendon autograft compared with patellar tendon-bone autograft for anterior cruciate ligament reconstruction. A randomized study with two-year follow-up. Am J Sports Med.

[B10] Beynnon BD, Johnson RJ, Fleming BC, Kannus P, Kaplan M, Samani J, Renström P (2002). Anterior cruciate ligament replacement: comparison of bone-patellar tendon-bone grafts with two-strand hamstring grafts. A prospective, randomized study. J Bone Joint Surg Am.

[B11] Ejerhed L, Kartus J, Sernert N, Köhler K, Karlsson J (2003). Patellar tendon or semitendinosus tendon autografts for anterior cruciate ligament reconstruction? A prospective randomized study with a two-year follow-up. Am J Sports Med.

[B12] Eriksson K, Anderberg P, Hamberg P, Löfgren AC, Bredenberg M, Westman I, Wredmark T (2001). A comparison of quadruple semitendinosus and patellar tendon grafts in reconstruction of the anterior cruciate ligament. J Bone Joint Surg Br.

[B13] Jansson KA, Linko E, Sandelin J, Harilainen A (2003). A prospective randomized study of patellar versus hamstring tendon autografts for anterior cruciate ligament reconstruction. Am J Sports Med.

[B14] O'Neill DB (1996). Arthroscopically assisted reconstruction of the anterior cruciate ligament. A prospective randomized analysis of three techniques. J Bone Joint Surg Am.

[B15] Shaieb MD, Kan DM, Chang SK, Marumoto JM, Richardson AB (2002). A prospective randomized comparison of patellar tendon versus semitendinosus and gracilis tendon autografts for anterior cruciate ligament reconstruction. Am J Sports Med.

[B16] Brandsson S, Faxen E, Eriksson BI, Swärd L, Lundin O, Karlsson J (1999). Reconstruction of the anterior cruciate ligament: comparison of outside-in and all-inside techniques. Br J Sports Med.

[B17] Gerich TG, Lattermann C, Fremerey RW, Zeichen J, Lobenhoffer HP (1997). One-versus two-incision technique for anterior cruciate ligament reconstruction with patellar tendon graft. Results on early rehabilitation and stability. Knee Surg Sports Traumatol Arthrosc.

[B18] Reat JF, Lintner DM (1997). One-versus two-incision ACL reconstruction. A prospective, randomised study. Am J Knee Surg.

[B19] Daniel DM, Stone ML, Daniel DM, Akeson WA, O'Connor JJ (1990). Case studies. Knee ligaments: Structure, Function, Injury, and Repair.

[B20] Harner CD, Fu FH, Irrgang JJ, Vogrin TM (2001). Anterior and posterior cruciate ligament reconstruction in the new millennium: a global perspective. Knee Surg Sports Traumatol Arthrosc.

[B21] Appel H (1970). Late results after meniscectomy in the knee joint. A clinical and roentgenologic follow-up investigation. Acta Orthop Scand.

[B22] Bolano LE, Grana WA (1993). Isolated arthroscopic partial meniscectomy. Am J Sports Med.

[B23] Johnson RJ, Kettelkamp DB, Clark W, Leaverton P (1974). Factors affecting late results after meniscectomy. J Bone Joint Surg.

[B24] McDaniel WJ, Dameron TB (1980). Untreated ruptures of the anterior cruciate ligament: A follow-up study. J Bone Joint Surg.

[B25] Aglietti P, Zaccherotti G, De Biase P, Taddei I (1994). A comparison between medial meniscus repair, partial meniscectomy, and normal meniscus in anterior cruciate ligament reconstructed knees. Clin Orthop.

[B26] Sherman MF, Warren RF, Marshall JL, Savatsky GJ (1988). Clinical and radiographic analysis of 127 anterior cruciate insufficient knees. Clin Orthop Rel Res.

[B27] Galway RD, Beaupré A, MacIntosh DL (1972). Pivot shift: a clinical sign of symptomatic anterior cruciate ligament insufficiency. J Bone Joint Surg Br.

[B28] McGuire DA (1992). The Paramax ACL Guide System Surgical Technique. Linvatec Concept Arthroscopy, 11311 Concept Boulevard Largo, Florida 34643.

[B29] Anderson AF, Fu FH, Harner CD, Vince KG (1994). Rating Scales. Knee Surgery.

[B30] Irrgang JJ, Ho H, Harner CD, Fu FH (1996). Use of the international knee documentation committee guidelines to assess outcome following anterior cruciate ligament reconstruction. AOSSM Speciality Day 30–1.

[B31] Tegner Y, Lysholm J, Gillquist J (1985). Rating systems in the evaluation system of knee surgery. Clin Orthop.

[B32] Hoher J, Munster A, Klein J, Eypasch E, Tiling T (1995). Validation and application of a subjective knee questionnaire. Knee Surg Sports Traumatol Arthrosc.

[B33] Bach BR, Warren JF, Flynn WM, Kroll M, Wickiewiecz TL (1990). Arthrometric evaluation of the knees that have a torn anterior cruciate ligament. J Bone Joint Surg Am.

[B34] Kellgren JH, Lawrence JS (1957). Radiological assessment of osteoarthritis. Ann Rheum Dis.

[B35] Fairbank TJ (1984). Knee joint changes after meniscectomy. J Bone Joint Surg.

[B36] Lohmander LS, Roos H (1994). Knee ligament injury, surgery and osteoarthrosis. Truth or consequences? Review. Acta Orthop Scand.

[B37] Jomha NM, Pinczewski LA, Clingeleffer A, Otto DD (1999). Arthroscopic reconstruction of the anterior cruciate ligament with patellar-tendon autograft and interference screw fixation. J Bone Joint Surg Br.

[B38] Donaldson WF, Warren RF, Wickiewicz T (1985). A comparison of acute anterior cruciate ligament examinations. Initial versus examination under anaesthesia. Am J Sports Med.

[B39] Hardacker WT, Garrett WE, Bassett FH (1990). Evaluation of acute traumatic haemarthrosis of the knee joint. South Med J.

[B40] Learmonth DJA (1991). Incidence and diagnosis of anterior cruciate ligament injuries in the accident and emergency department. Injury.

[B41] Daniel DM, Stone ML, Daniel DM, Akeson WA, O'Connor JJ (1990). KT-1000 anterior-posterior displacement measurements. Knee ligaments: Structure, Function, Injury and Repair.

[B42] Good L, Askew MJ, Boom A, Melby A (1993). Kinematic in vitro comparison between the normal knee and two techniques for reconstruction of the anterior cruciate ligament. Clin Biomech.

[B43] Good L, Askew MJ, Boom A, Melby A (1993). Kinematic in vitro comparison between the normal knee and two techniques for reconstruction of the anterior cruciate ligament. Clin Biomech.

[B44] Heerwaarden van RJ, Stellinga D, Frudiger AJ (1996). Effect of pre-tension on reconstructions of the anterior cruciate ligament with a Dacron prosthesis. A retrospective study. Knee Surg Sports Traumatol Arthrosc.

[B45] Ferretti A, Conteduca F, DeCarli A, Fontana M, Mariani PP (1991). Osteoarthritis of the knee after ACL reconstruction. Int Orthop.

[B46] Jørgensen U, Sonne-Holm U, Lauridsen F, Rosenklint A (1987). Long-term follow-up of meniscectomy in athletes. A prospective longitudinal study. J Bone Joint Surg Br.

[B47] Lynch M, Henning CE, Feagin JA (1981). Osteoarthritis in the ACL deficient knee. The crucial ligaments: diagnosis and treatment of ligamentous injuries about the knee.

[B48] Rangger C, Klestil T, Gloetzer W, Kemmler G, Benedetto KP (1995). Osteoarthritis after arthroscopic partial meniscectomy. Am J Sports Med.

[B49] Satku K, Kumar JP, Ngoi SS (1986). Anterior cruciate ligament injuries: to counsel or operate?. J Bone Joint Surg Br.

[B50] Fukubayashi T, Kurosawa H (1980). The contact area and pressure distribution pattern of the knee: a study of normal and osteoarthritic knee joints. Acta Orthop Scand.

[B51] O'Brien WR (1993). Degenerative arthritis of the knee following anterior cruciate ligament injury: role of the meniscus. Sports Med Arthrosc Rev.

[B52] Sommerlath K, Lysholm J, Gillquist J (1991). The long-term course after treatment of acute anterior cruciate ligament ruptures: a 9 to 16 year follow-up. Am J Sports Med.

[B53] Fitzgibbons RE, Shelbourne KD (1995). 'Aggressive' nontreatment of lateral meniscal tears seen during anterior cruciate ligament reconstruction. Am J Sports Med.

[B54] Allen PR, Denham RA, Swann AV (1984). Late degenerative changes after meniscectomy. Factors affecting the knee after operation. J Bone Joint Surg Br.

[B55] Felson DT (1993). The course of osteoarthritis and factors that affect it. Review. Rheum Dis Clin North Am.

[B56] Roos H, Adalberth T, Dahlberg L, Lohmander LS (1994). Post-traumatic osteoarthritis of the knee, disease progress after injury to the anterior cruciate ligament or meniscus: the influence of time and age. Trans Orthop Res Soc.

[B57] Scott WW, Lethbridge-Cejku M, Reichle R, Wigley FM, Tobin JD, Hochberg MC (1993). Reliability of grading scales for individual radiographic features of osteoarthritis of the knee. The Baltimore longitudinal study of aging atlas of knee osteoarthritis. Invest Radiol.

[B58] Doherty M, Watt I, Dieppe P (1983). Influence of primary generalised osteoarthritis on development of secondary osteoarthritis. Lancet.

[B59] Vikkula M, Metsaranta M, Ala-Kokko L (1994). Type II collagen mutations in rare and common cartilage diseases. Review. Ann Med.

[B60] Williams CJ, Jimenez SA (1993). Heredity, genes and osteoarthritis. Review. Rheum Dis Clin North Am.

[B61] Agneskirchner JD, Burkart A, Imhoff AB (2002). Axis deviation, cartilage damage and cruciate ligament rupture–concomitant interventions in replacement of the anterior cruciate ligament. Unfallchirurg.

[B62] Saxon L, Finch C, Bass S (1999). Sports participation, sports injuries and osteoarthritis implications for prevention. Sports Med.

[B63] Arnold JA, Coker TP, Heaton LM, Park JP, Harris WD (1979). Natural history of anterior cruciate tears. Am J Sports Med.

[B64] Jacobsen K (1977). Osteoarthrosis following insufficiency of the cruciate ligaments in man. A clinical study. Acta Orthop Scand.

[B65] Neyret P, Donell ST, Dejour H (1993). Results of partial meniscectomy related to the state of the anterior cruciate ligament. Review at 20 to 35 years. J Bone Joint Surg Br.

[B66] Sahlstrom A, Johnell O, Redlund-Johnell I (1997). The natural course of arthrosis of the knee. Clin Orthop Relat Res.

[B67] Spector TD, Dacre JE, Harris PA, Huskisson EC (1992). Radiological progression of osteoarthritis: an 11 year follow up study of the knee. Ann Rheum Dis.

[B68] Gillquist J, Messner K (1999). Anterior cruciate ligament reconstruction and the long term incidence of gonarthrosis. Sports Med.

[B69] Roos H (1994). Anterior cruciate ligament injuries and soccer – an incompatible combination?. Exercise, knee injury and arthrosis (Thesis) University Hospital, Lund.

[B70] Sommerlath K (1989). The importance of meniscus in unstable knees. A comparative study. Am J Sports Med.

[B71] Akeson WH, Daniel DM, Akeson WA, O'Connor JJ (1990). The response of ligaments to stress modulation and overview of the ligament healing response. Knee ligaments: Structure, Function, Injury, and Repair.

[B72] Amiel D, Kuiper S, Daniel DM, Akeson WA, O'Connor JJ (1990). Experimental studies on anterior cruciate ligament grafts: Histology and biochemistry. Knee ligaments: Structure, Function, Injury, and Repair.

[B73] Sachs RA, Reznik A, Daniel D, Stone ML, Daniel DM, Akeson WA, O'Connor JJ (1990). Complications of the knee ligament surgery. Knee ligaments: Structure, Function, Injury, and Repair.

